# Effect of anlotinib combined with ticeorgio for recurrent nasopharyngeal carcinoma: a case report

**DOI:** 10.3389/fphar.2023.1166809

**Published:** 2023-07-14

**Authors:** Jiwei Mao, Wanli Ye, Dongping Wu, Jianjiang Liu, Ting Li, Weili Ma, Yang Zhou

**Affiliations:** ^1^ Department of Radiation Oncology, Shaoxing People’s Hospital, Shaoxing, Zhejiang Province, China; ^2^ Department of Radiology, Shaoxing People’s Hospital, Shaoxing, Zhejiang Province, China; ^3^ Emergency Department, Shaoxing People’s Hospital, Shaoxing, Zhejiang Province, China

**Keywords:** anlotinib, combined therapy, recurrent nasopharyngeal carcinoma, ticeorgio, side effects of re-radiotherapy, S-1

## Abstract

For patients with locally unresectable recurrent nasopharyngeal carcinoma who relapsed after 2 years of radiotherapy, re-radiotherapy is also the preferred treatment. However, for patients relapsed within 2 years, the use of re-radiotherapy would be greatly limited by its adverse effects. Consequently, finding a new strategy to prolong the time of re-radiotherapy for locally recurrent nasopharyngeal carcinoma is very necessary to reduce the related side effects and improve the curative effect. Anlotinib is an orally available small molecule multi-target tyrosine kinase inhibitor that primarily inhibits VEGFR2/3, FGFR1–4, PDGFR α/β, c-Kit, and Ret. However, whether recurrent nasopharyngeal carcinoma patients can be treated with anlotinib combined with ticeorgio (also called S-1) remains unknown. Herein, we report a nasopharyngeal carcinoma patient with local recurrence after radical radiotherapy who benefited from combination treatment of anlotinib with ticeorgio.

## Introduction

Nasopharyngeal carcinoma (NPC) is a malignant tumor arising from the nasopharyngeal epithelium. With an estimated 133,354 new cases and 80,008 associated deaths worldwide, NPC is a relatively rare disease, representing approximately 0.7% of cancer diagnoses and 0.8% of cancer-related deaths (Sung et al., 2021). The distribution of NPC has strong regional characteristics, with approximately half of NPC cases arising in China (Chen et al., 2019). Induction chemotherapy followed by concurrent chemoradiotherapy is the standard treatment for locoregionally advanced NPC. For patients who cannot tolerate or are unwilling to receive chemotherapy, radiotherapy combined with nimotuzumab can be an option in China (Radiation Oncology Physicians Branch of Chinese Medical Doctor Association ROBoCMA, 2022).

With the increased application of intensity modulated radiotherapy (IMRT) and comprehensive treatment plans, the 5-year local and regional relapse-free survival rates in patients with newly diagnosed nonmetastatic NPC are between 83.0% and 91.8% and between 91.0% and 96.4%, respectively (He, 2021); however, it has been reported that between 10% and 20% of patients still experience local recurrence (Li et al., 2018; Lee et al., 2019). According to the National Comprehensive Cancer Network guidelines for unresectable head and neck cancer with locoregional recurrence previously treated with radiotherapy, radiotherapy is the recommended treatment for unresectable locally recurrent NPC. Nevertheless, incidences of reirradiation-related grade 3–4 late toxic effects after salvage IMRT remain between 34% and 70% (Han et al., 2012; Hua et al., 2012). These toxic effects can be particularly pronounced within 2 years after primary radiotherapy. Thus, choosing an appropriate treatment strategy for patients with unresectable NPC who have relapsed within 2 years after radiotherapy is a challenge for oncologists.

Anlotinib is an orally available small molecule multi-target tyrosine kinase inhibitor that primarily inhibits VEGFR2/3, FGFR1–4, PDGFR α/β, c-Kit, and Ret and has been widely used in the clinic (Shen et al., 2018). A recent study reported that anlotinib combined with ticeorgio showed favorable efficacy for advanced NPC patients who had failed first-line treatment (Yang and Chen, 2022). However, whether patients with advanced recurrent NPC can be treated with anlotinib combined with ticeorgio remains unknown. Herein, we report the case of a patient with locally recurrent NPC after radical radiotherapy who benefited from anlotinib in combination with ticeorgio.

## Case presentation

**FIGURE 1:**
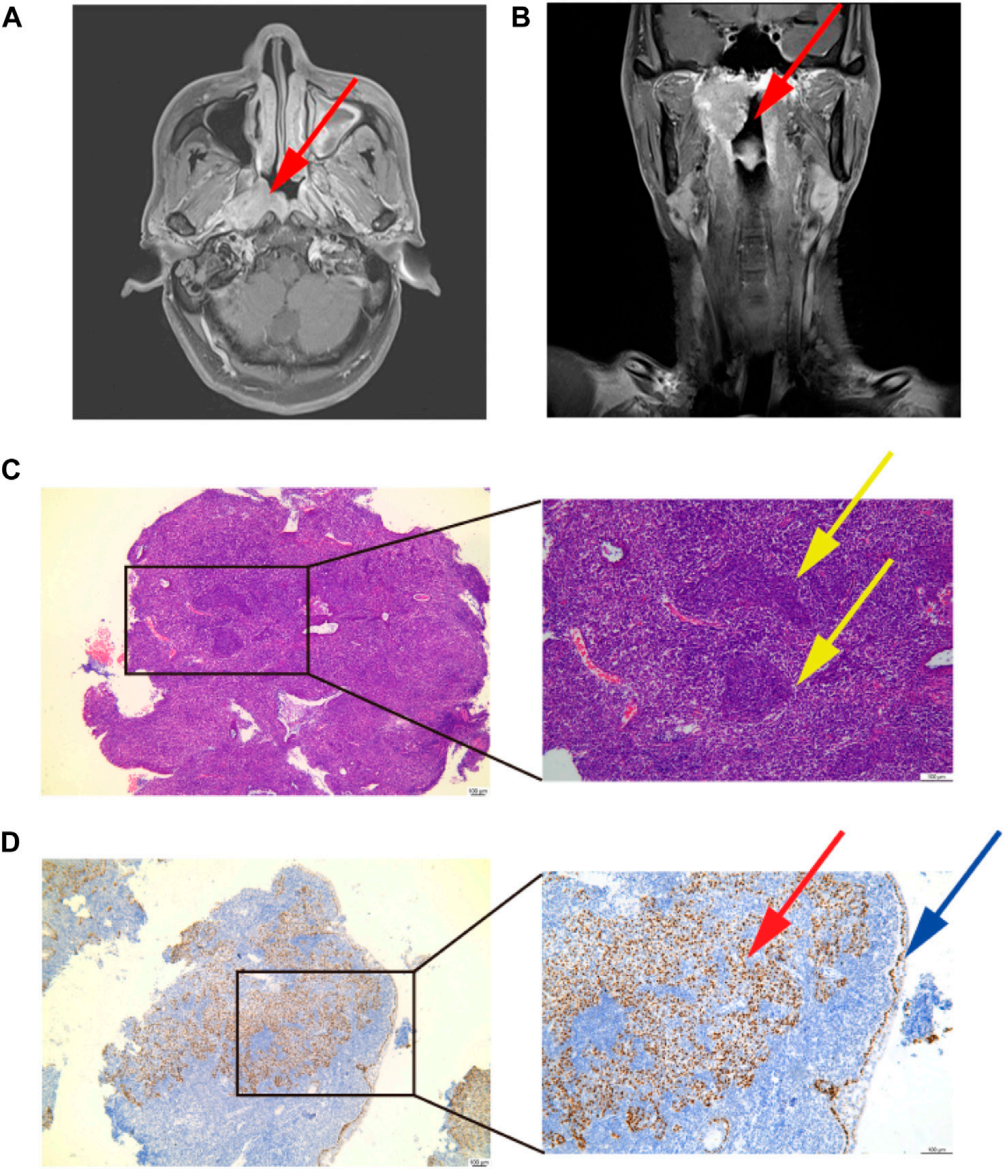
Diagnosis of nasopharynx cancer. **(A,B)** Diagnostic imaging result. A mass on the posterior wall of the nasopharynx: the red arrow points to. **(C)** Image of pathological diagnosis. Magnification; ×40 and×100. A paraffin section of the mass was stained with HE, showing obvious tumor nests, oval nuclei: the yellow arrow points to. **(D)** P63 immunohistochemical staining. Magnification;×40 and ×100. A paraffin section of the mass was stained with P63, a large number of P63-positive cells can be seen under the basement membrane, squamous cell carcinoma is positive for P63: the red arrow points to. P63-positive cells indicated by blue arrows are the basement membrane.

A previously healthy 60-year-old man visited the ENT Department owing to right ear tinnitus and stuffy discomfort for 20 days. The first doctor performed a contrast-enhanced magnetic resonance scan of the nasopharynx, which showed a mass on the posterior wall of the nasopharynx, then we reexamined a 3-T contrast-enhanced MRI (Figures 1A, B **)**. The patient was subsequently diagnosed with NPC by pathological biopsy (Figures 1C, D **)**. The pathological classification was non-keratinizing undifferentiated carcinoma, and the TNM staging was stage III (T3N2M0) according to the eighth edition of the American Joint Committee on Cancer guidelines.

**FIGURE 2:**
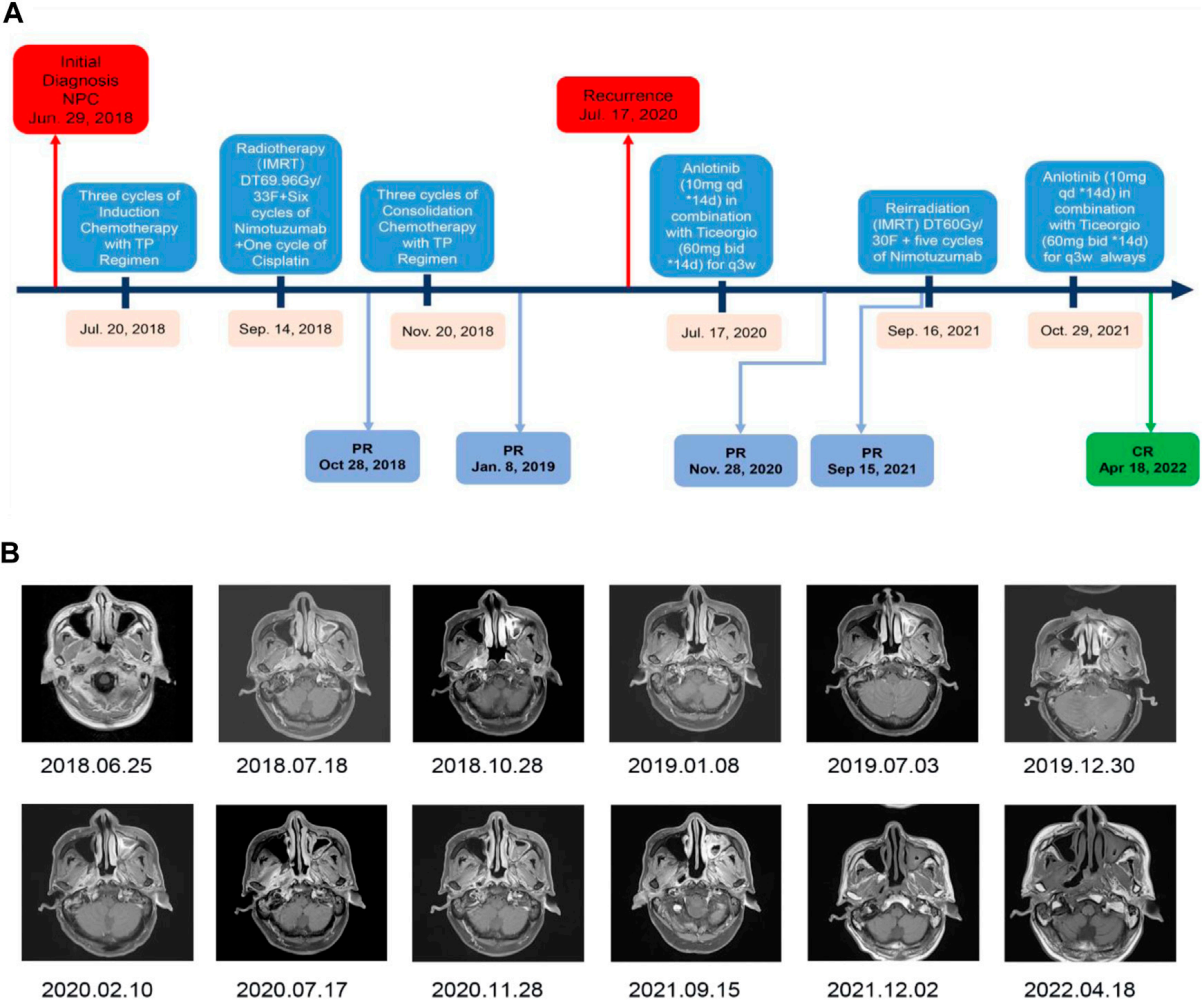
Timeline of treatment and radiographic responses. **(A)** Timeline of treatment. **(B)** Imaging results showing regression of the lesion and demonstrating the effectiveness of the anlotinib combined with ticeorgio regimen.

The patient was then referred to the Radiotherapy Department and received three cycles of induction chemotherapy with the TP regimen (paclitaxel [270 mg] + cisplatin [135 mg]), which resulted in partial response (PR). Then, treatment was performed with radical radiotherapy at a dose delivered to the gross tumor volume of the nasopharynx (PGTVnx) and gross tumor volume of the lymph nodes (PGTVnd) of at least 6,996 cGy in 33 fractions, with 212 cGy daily five times per week; targeted therapy with nimotuzumab once weekly for 6 weeks and one cycle of cisplatin chemotherapy were performed concurrently. After radiotherapy, three cycles of consolidation TP chemotherapy (paclitaxel [270 mg] + cisplatin [135 mg]) were performed. All treatments resulted in PR (Figure 2A). During the timely periodic follow-up checks, residual tumor foci decreased further in size, and the final assessment of efficacy was cCR (Figure 2B 2019.07.03). In the following 5 months, there is no sign of recurrence discovered. However, the result of the sixth month’s follow-up examination revealed parapharyngeal lymph node exhibited a trend toward increasing size (Figure 2B 2020.02.10). Five months later the patient developed a slight restriction of mouth opening. And a subsequent MR (Figure 2B 2020.07.17) scan showed further enlargement of the parapharyngeal lymph nodes, leading to invasion of the parapharyngeal space and the lateral pterygoid muscle. According to the Expert consensus on the treatment of recurrent nasopharyngeal carcinoma, recurrent NPC is defined as 6 months after radical treatment of the first diagnosis of nasopharyngeal carcinoma, during which the tumour tissue has reached cCR or pCR, followed by a recurrence of tumour growth (Lin et al., 2018). Recurrence was defined on the basis of radiographic and clinical findings.

In conclusion, 18 months after completing treatment, the patient was diagnosed with recurrent NPC in the parapharyngeal space by contrast-enhanced magnetic resonance imaging of the nasopharynx. Thereafter, the patient received anlotinib (10 mg qd for 14 days) combined with ticeorgio (60 mg bid for 14 days, based on 60 mg per dose for body surface area >1.5 m^2^, twice a day) every 3 weeks, drugs administration were performed daily by an oral rout, which resulted in PR. Common adverse drug reactions of anlotinib and ticeorgio include hypertension, proteinuria, nausea, vomiting, liver and kidney function injury, bone marrow suppression, fatigue, and so on. Despite that, the actual toxic effects were just mild fatigue during the co-medications period for this patient. Considering the effectiveness of the combination therapy regimen and its mild side effects, we tried to use this treatment as much as possible to gain time and space for a further re-radiotherapy. After 14 months of treatment with anlotinib and ticeorgio, the patient received re-radiotherapy at a dose delivered to the recurrent gross tumor volume of the nasopharynx (PTV) of at least 6,000 cGy in 30 fractions, with 200 cGy daily five times per week concurrently with nimotuzumab once weekly for 6 weeks. Maintenance treatment with anlotinib (10 mg qd for 14 days) combined with ticeorgio (60 mg bid for 14 days) every 3 weeks was continued when re-radiotherapy had finished. The lesion was evaluated as complete response on 2 December 2021. As of 28 April 2022, the lesion remains stable and no residual cancer has been found on imaging.

## Discussion

This study investigated whether anlotinib combined with ticeorgio could be an effective regimen for patients with locally recurrent NPC who had received comprehensive treatments, including induction chemotherapy plus concurrent chemoradiotherapy, concurrent targeted therapy, and consolidation chemotherapy after radical radiotherapy. Anlotinib is a novel anti-angiogenesis drug that was designed to primarily inhibit VEGFR2/3, FGFR1–4, PDGFR α/β, c-Kit, and Ret, and it shows broad-spectrum antitumor potential in advanced refractory solid tumors (Sun et al., 2016) and seemed to be a promising therapeutic option for recurrent or metastatic NPC with acceptable toxicity profiles (Cai et al., 2020). Recently, anlotinib has been increasingly used in clinical practice for advanced tumors, and not only as an individual agent (Sun Y. et al., 2021; Cheng et al., 2021; Chi et al., 2021; Cui et al., 2021; Zhu et al., 2021) but also combined with immunotherapy (Han et al., 2021; Wang et al., 2021; Zhang et al., 2021; Su et al., 2022; Xu et al., 2022), radiofrequency ablation (Zhou et al., 2021), radiotherapy (He et al., 2021), and chemotherapy (Sun W. et al., 2021; She et al., 2021; Xiang et al., 2021). These studies have all shown promising efficacy and safety data for anlotinib. Ticeorgio is the Chinese version of S-1, which is a novel oral fluoropyrimidine agent containing tegafur, gimeracil and oteracil potassium (Oki et al., 2016; Zheng et al., 2019). Clinical trials showed that ticeorgio plus gemcitabine or nedaplatin have provided a satisfactory and safe clinical activity for patients with recurrent and metastatic NPC after platinum-containing chemotherapy failed (Peng et al., 2016; Peng et al., 2017). These studies suggested that anlotinib and ticeorgio are likely to be effective in recurrent metastatic nasopharyngeal carcinoma, meanwhile the adverse effects are tolerable. Notably, one clinical trial showed that anlotinib combined with ticeorgio had favorable efficacy for advanced NPC patients who had failed first-line treatment (Yang and Chen, 2022). However, there are no reported cases of locally recurrent NPC treated with anlotinib, not to mention the combination of anlotinib and ticeorgio.

In this case, the patient experienced recurrence within 2 years after radical radiotherapy. According to the National Comprehensive Cancer Network guidelines for head and neck cancer with locoregional recurrence previously treated with radiotherapy, surgical resection is the preferred approach for resectable recurrent tumors. However, given that the recurrent tumor was located within the parapharyngeal space, surgical treatment would have been quite difficult, with the potential for serious complications. Thus, the patient was not a candidate for surgery. For locally unresectable recurrent NPC, re-radiotherapy is the preferred choice. However, if the patient had received radiotherapy again in such a short period of time, there would have been a high probability of serious and even lethal toxic side effects. It is important to extend the interval between re-radiotherapy for the patient. Therefor platinum-based systemic chemotherapy is a good choice to effectively control tumor progression. Due to unpleasant side effects from previous chemotherapy, the patient was resistant to platinum-based chemotherapy. Given the patient’s wishes, the above reasons and in accordance with previous reports, we selected the anlotinib plus ticeorgio regimen to extend the interval between the two radiotherapy courses. Therefore, in this case, the patient received anlotinib (10 mg once daily) and ticeorgio (60 mg twice daily) for 2 weeks on with 1 week off. The patient responded well to this therapy, with the lesion dramatically decreasing in size and forming tumor cavitation after 4 months of therapy. Then the patient continued to be treated with anlotinib and ticeorgio for 10 months, which resulted in PR and further enlargement of the tumor cavity. Tumor cavitation is an independent predictor of better PFS in NPC (Chen et al., 2020). During anlotinib therapy, tumor cavitation occurred in our patient and grew over treatment. However, effective candidate biomarkers for anlotinib remain unclear, and further investigation is warranted.

After 14 months of combined treatment with anlotinib and ticeorgio, the patient received re-radiotherapy with concurrent targeted sensitization therapy with nimotuzumab. After re-radiotherapy, the patient continued treatment with anlotinib (10 mg qd for 14 days) combined with ticeorgio (60 mg bid for 14 days) every 3 weeks. Subsequently, the lesion was evaluated as complete response (CR) on 2 December 2021. As of 28 April 2022, the lesion has remained stable, with no signs of residual cancer on imaging. Ongoing follow-up will continue, and we will look for opportunities to provide further updates on the patient’s condition.

## Conclusion

Overall, this case highlights the potential efficacy of anlotinib combined with ticeorgio for recurrent refractory NPC. In this patient, anlotinib plus ticeorgio had a miraculous effect on recurrent refractory NPC. However, testing of this regimen has had some limitations, such as small case samples. In future studies, we aim to further explore this option in larger cohorts.

## Data Availability

The original contributions presented in the study are included in the article/supplementary material, further inquiries can be directed to the corresponding author.
